# Sudden unexpected physicians' death during performing professional duties: increasing social and public health problem

**DOI:** 10.13075/ijomeh.1896.02808

**Published:** 2026

**Authors:** Jolanta Bilińska, Joanna Ruszkowska, Hussain Jafri, Wojciech Drygas

**Affiliations:** 1 The President Stanislaw Wojciechowski University of Kalisz, Institute for Patients' Safety Research, Kalisz, Poland; 2 World Patients Alliance, European Region, Washington, DC, USA; 3 Medical University of Lodz, Department of Preventive Medicine, Łódź, Poland

**Keywords:** suicide, physicians, sudden death, work overload, medical doctors, *karoshi*

## Abstract

A work-related death from sudden causes among physicians is not a new problem although still not enough explored in the scientific literature. Estimat ing the scale of the phenomenon is important from a social and public health point of view. Thus, the aim of this paper is to gather the current state of knowledge on the frequency and the main causes of unexpected sudden causes of death within physicians' community. The authors of the article reviewed medical databases on publications in English concerning the topic released in this century. The cases of sudden death, work-overload death including those resulting from suicide and incidences of direct violence from other individuals while performing professional duties were analyzed. It turns out that the problem appears regardless of the region of the world and has been increasing in frequency in recent times. Some medical specialties, i.e., surgery, psychiatry, anesthesiology, intensive care and emergency medicine, seem to carry significantly higher risk than others. In addition to the conclusions from literature review, also letters to the editors and mass-media reports from different parts of the world indicate that the problem is alarming and requires more specific research and urgent solutions.

## Highlights

- Physicians experience huge work overload results in sudden death or suicide.
- Physician's death is often interesting for mass-media but not for scientific world.
- Sudden unexpected physicians' death seems to be a global problem.

*One skilled physician is worth a hundred warriors*

Homer (8th century BC)

## INTRODUCTION

A death from sudden causes among physicians, although tragic, unfortunately is not a new phenomenon. However, it must be admitted that in the scientific literature there is little information on this subject, as if it were a kind of taboo topic.

In history, one of the first known deaths related to the professional duties was the case of Professor Johann Wirsung, the one who had discovered the main pancreatic duct and was murdered by his student in 1643 in Padua, Italy [1,2]. Two centuries later in New Zealand between 1840 and 1910, the leading causes of doctors' mortality became a topic of scientific interest, with violence and suicide ranking very high [3].

It appears that there has not been much of a change in the situation over the years. The literature shows that the suicide rates in American physicians in the 1970s were 2 times higher than in the general population, with women having 4 times higher rates [4,5]. Worse still, in the 1990s a new harmful phenomenon had also been noticed among medical professionals: *karoshi* – sudden death from work overload, although the first *karoshi* case was reported many years earlier in 1969 in Japan [6,7].

Nowadays, reports of sudden physician deaths can be found on the front pages of newspapers. Some of these cases are unclear, dubious and probably politically motivated, like the death of Sergey Maximishin, the physician who was taking care of poisoned Alexey Navalny in the Omsk emergency hospital [8]. Other media reports are visible because they concern popular and important people, such as recent cardiac death of 53-year-old doctor Carles Minarro Garcia, a member of FC Barcelona medical team [9,10]. Obviously, it can be assumed that many cases of medical doctors' deaths resulting from the performance of professional duties still remain unnoticed. It seems that the scientific world is also not interested enough to estimate the scale and details of the disquieting phenomenon.

World Health Organization defines sudden death as a non-aggressive and mysterious death occurring within 24 h of the beginning of symptoms [11]. But looking through the prism of public health consequences, we find this definition insufficiently precise and gives an incomplete picture of physicians' deaths. Taking into account changing times, particularly in the post-COVID era, as well as the threat posed by a disturbed work–life balance and the substantial responsibility placed on medical professionals, sudden physician deaths related to the performance of professional duties may be defined as unexpected fatal events occurring within a relatively short timeframe, often without preceding significant symptoms. This definition includes deaths resulting from work-overload-related suicide (*karojisatsu*) and direct violence perpetrated by other individuals (e.g., patients, patients' family members, or visitors).

The authors believe that estimating the scale of the phenomenon is important not only from cognitive reasons, but also from occupational medicine and public health point of view. Thus, the aim of this paper is to gather the current state of knowledge on the frequency and the main reasons for unexpected sudden causes of death concerning physicians' community.

## METHODS

In order to carry out the review, the most popular electronic scientific databases were used to identify the articles in English published in this century using the following keywords: sudden death, suicide, work overload, karoshi, physicians, medical doctors and health professionals. The publications were reviewed, and the relevant ones were cited. The data for this narrative review article were extracted from medical databases, official documents, Medical Bulletin, Cochrane library, Web of Science, The Lancet, PubMed, etc. The authors decided to choose a long period of time 2000–2025 in order to include most important but scares scientific reports from Asia, Europe, USA, Canada and other parts of the World.

## RESULTS

In medical literature about physicians' deaths on duty, the information that is available is limited and sparse. Table 1 provides key information on the relevant papers reviewed for this study. Until now, most of the scientific reports and literature on this topic are from China, South Korea, India, Pakistan, Japan, USA and Australia. In the last decade occupational deaths among physicians have become a challenging problem in hospital management [12]. The causes of these deaths include high workload, disproportionate allocation of medical resources, work related stress, excessive burden of academic research and the difficulty of being your own doctor [12].

**Table 1:** Selected studies on physicians' occupational deaths published in 2000–2025 in Asia, Europe, and North America

Study	Objective and country	Study population	Results	Reasons
Liang et al., 2021 [13]	to investigate the characteristics and relationship between abnormal doctors deaths and workload in Chinese hospitals in 2007–2020 (China)	surgery, anaesthesiology, internal medicine, orthopaedics	51 doctors died from cardiogenic diseases; 27 doctors committed suicide; 12 doctors died of brain derived diseases	high intensity work, extremely long working hours, day night reversal, continuous work without rest, extremely heavy physical work, work with high mental pressure
Xu et al., 2020 [15]	to summarize the cases of doctors work overload deaths in 2007–2018 (China)	anaesthesiology, orthopaedics	anaesthesiologist: 22 died; orthopaedics: 20 died	reasons of death: work overload, burnout, decreased patient–physician trust; cause of sudden deaths: aortic dissection, cancer, cerebral haemorrhage, heart attack
Kang et al., 2021 [16]	letter of editor on sudden deaths amongst young and middle aged doctors in China (China)	physicians' deaths from the departments of dermatology, breast surgery, ophthalmology, liver surgery, laboratory, transplantation and health statistics	8 doctors (ageing 41–57 years), mostly male, suffered sudden deaths in 2 months	doctors worked continuously >24 h, 50% of them >36 h, about 20% >48 h; average weekly working time >40 h
Shin et al., 2022 [80]	to evaluate the health status of healthcare workers in Korea in 2002–2017 (Korea)	doctors, nurses	2600 died during study period, out of which 2077 were doctors; doctors had higher prevalence of high blood pressure, asthma and high risk for migraine, stroke, epilepsy and depression	doctors deaths were related to medical reasons, accident suicide; occupational characteristics of healthcare workers
Song et al., 2015. [14]	first academic report on the sudden deaths of Chinese physicians in 2008–2015 (China)	physicians who died were from the departments of anaesthesiology, surgery, internal medicine and emergency	29 physicians aged 26–55 years died	the majority of reported deaths from heart related causes such as cardiac arrhythmia
Sood et al., 2022 [28]	to assess the risk of suicide and self-harm among physician and non-physicians in 1990–2016 (Canada)	35 989 physicians, 6 585 197 non-physicians	risks of suicide and self-harm are similar in both populations	key risk factors such a mental health disorder could prompt the high index of concerns
Kavaliauskas et al., 2024 [24]	to evaluate suicidality and factors associated with high suicide risk among physicians and residents (Lithuania)	physicians, residents	lifetime suicide risk was found among 30.4% of participants; 11.4% reported current and past suicidal ideation; 2.5% reported previous suicide attempt	main suicide risk factors: poor mental health, work related stressors and lack of long term relationships

### Sudden unexpected physicians' deaths: global or regional problem?

Sudden deaths among physicians are not rare, but not well documented in reports and scientific documents. The media mostly covers incidents in hospitals and outpatient clinics involving physicians who died on duty. Reports from China reveal that physicians are faced by unusual deaths, including homicide or work overload [13] and data from 2008 and 2015 show an escalation of sudden deaths among Chinese physicians.

This first academic report in China on sudden deaths among physicians highlights work overload to be the most alarming problem that physicians have to deal with [14]. Liang et al. [13] examined relationship between abnormal death and physician work burden in 2007–2020. The report intended to provide information to the policy makers to take remedial measures related to sudden deaths. They collected data about 207 deaths out of which the majority died from work overload or sudden death. These physicians mainly worked as surgeons, anaesthesiologists, internists and orthopaedic doctors.

Another study on 110 work overload related deaths of Chinese physicians in 2007–2018 reveals that the majority were male from the ages of 34–48 years, working in the department of anaesthesiology in first-tier cities, followed by orthopaedics [15]. The 2018 white paper by Chinese Medical Association on the practice of Chinese physicians reports that on average physicians in China exceed the 40-hour weekly limits. Moreover, >75% of the physicians are not able to take legal annual leave, underscoring the fact that their right to rest is not protected [16].

The Lancet, one of the most valued medical scientific journals, drew attention to the case of a 32-year-old ophthalmologist, who died suddenly after working with fever for 6 days in a hospital in Beijing [17]. It emphasizes the issue of physicians' wellbeing in low- and middle-income countries (LMIC) countries. Most of these deaths result from heavy workload.

Sudden deaths amongst physicians have largely been reported in China over the past year on social media, however, little is reported in clinical reports. Gao et al. [18] have brought to attention that physicians from surgery and emergency medicine departments are worst affected. They further reported that Chinese doctors work under different pressures such as low salary, violence against doctors, significant expectations at work placed on them [18]. Moreover, work overload, staff shortage, long working hours and high patient turnover are also reported as reasons for such deaths with medical causes mentioned as heart diseases, cardiac arrhythmias and epilepsy [19].

The number of hospital beds *per capita* in China is 3.9 beds/1000 people, which is comparable with developed countries like Australia. However, when it comes to hospital doctors in China, the numbers fall behind at 70% of the Australian level [20]. There were 97 million physicians in China by the end 2014, but it is not enough to cover all patients needs in the country [14].

Although a Chinese survey shows that half of the doctors worked at least 1 night shift/week, the overall workload was much higher. Around 80% reported to have only getting a 30 min break during the day with most did not even get 10 min for lunch. The respondents of the survey also complained of shifts of about 24 h, with half working close to 36 h, whereas roughly 20% respondents said that they routinely worked around 48 h [16].

### Suicide among physicians

Suicide is another important reason for sudden physicians' deaths. An estimated 300 physicians die by suicide per year, and rates may be rising. In the USA alone, the average number of physician suicides is 119 per year [21].

Some specialities can be at higher risk of suicide such as anaesthesiologist, psychiatrists, general practitioners and general surgeons [22]. The causes of these suicides are reported to be heavy workload, long shifts at work, unpredictable hours of work, work related stress and easy access to drugs [22]. Female doctors were at higher risk compared to males.

Albuquerque and Tulk [23] reported in 2019 that physicians have higher suicide rate as compared to the general population. In cross-sectional survey from the UK, nearly 8000 physicians who had a past or current regulatory complaints were significantly more likely to report suicidal ideation. There is a need to resolve this problem globally. The analysis of the literature shows that the physicians who died by suicide were less likely to be receiving mental health treatment than non-physicians who died by suicide.

Medical doctors and residents are exposed to many stressors in their daily work, which can lead to mental health problems. These pressures can cause psychological stress, anxiety and depression, and can also lead towards suicidal behaviour.

A study from Lithuania provides interesting insights into factors linked with high suicide risk among physicians. The research surveyed 685 doctors using an online questionnaire. The results of the survey showed lifetime suicide risk in 30.4% respondents, 11.4% reported suicidal planning, and 2.5% revealed a previous suicide attempt. This shows that nearly half of the physicians in the sample had experienced suicidal thoughts or behaviours at a high-risk level [24].

A 2016 Pakistani study also reported incidents of a medical student and a 27-year old physician committing suicides [25]. The suicide risks faced by Pakistan physicians included work overload, academic pressure, undetected depression, limited opportunities, financial and training challenges, family and social stress, junior–senior conflict, lack of emotional support, and insecurity created by violence and targeted attacks on doctors [25].

Physicians in India have 2.5 times more suicide risk than the general population. An Indian study reported 30 suicides in 2016–2019, out of which 18 were female and 12 male. More than 80% were <40 years old. The reasons mentioned for these suicides included depression, harassment, bullying, exam anxiety, burnout, mental health, marital discord [26].

However, suicide rate among physicians is not homogenous in all countries. For example, in some European countries suicide rates have decreased, probably due to the implementation of Working Time Directive by the EU [27], which is an excellent working time regulation for physicians. Similarly, the suicide rates in Canadian physicians are also not high and is comparable to the general population [28].

These examples show that improvements in the working conditions of physicians could bring down the suicide rates. Therefore, physicians who attempted to commit suicide as well as those with suicidal ideation could benefit from preventive strategies such as predictable working hours, short shifts, better communication, no sleep deprivation and identification of mental stress [21].

### Burnout among physicians: a global phenomenon?

Another complex and multifaceted problem is burnout because of workload. Burnout is a clinical condition characterized by physical and emotional exhaustion, depersonalisation, and a reduced sense of accomplishment. While burnout is not unique to clinicians, they are particularly susceptible to it. Burnout has been commonly observed in healthcare workers since 1970. It is mentioned in the literature that the phenomenon of burnout among doctors is a global phenomenon, however, the prevalence is geographically and speciality variable [29].

The Lancet report discloses that 78% of physicians experience burnout globally [17]. While analysing the reasons of premature physicians' deaths doctors' burnout cannot be ignored. A Chinese version of the *Maslach Burnout Inventory Human Services Survey* was conducted in 2019 with an objective to determine the factors related to physicians' burnout. The survey was mailed to 768 residents, of whom 563 completed it, giving a response rate of 73.3%. The results showed that physicians working in trauma surgery and outpatient specialties had a greater association with burnout [29].

Current literature also shows that 20% of medical residents met criteria for depression, while 74% met criteria for burnout [22]. The data highlights the need to avoid burnout situations at work by taking important steps such as physicians' self-awareness, gratification at work and understanding the risks at work.

### Doctors deaths during COVID-19 pandemics

The COVID-19 pandemics caused unexpected and very strong threat worldwide not only for patients but also for medical staff in many countries. Unfortunately, in the scientific literature and in official reports there are only scarce and mostly not precise information about doctors death due COVID pandemics. Ing et al. [30] revealed that on April 15, 2020 based on Google internet search 278 doctors died with COVID-19 infection with most reported physicians deaths in Italy, Iran, Philippines, Indo nesia, China, Spain, USA and UK. General practitioners, emergency room doctors and internal medicine specialists represented nearly 50% of all casualties. Median age of the physicians was 66 years and 90% were males. The authors emphasized that lack of personal protective equipment was cited as common cause of death. In their opinion consideration should be made to exclude older physicians from front-line work during pandemic. In the first period of pandemics (till July 13, 2020) about 23 000 deaths among patients have been reported in India, including 108 COVID-linked deaths among doctors. Over half of deaths occurred among general practitioners, and in 27% in doctors with surgical specialties [31]. The majority of deaths occurred <60 years of age. Polish reliable sources documented that due to COVID-19 altogether 558 healthcare workers died in January 2021–July 2022, including 200 doctors and 236 nurses [32]. The highest infection fatality rate (IFR) concerned the group of dentists, the lowest IFR value concerned physiotherapists. According to WHO assessment only between January 2020 and May 2021 80 000–180 000 health workers died from coronavirus disease as a result of working on the frontline battle with COVID pandemics [33]. Unfortunately, there is not possible to conclude from WHO reports how many doctors died as well as whether doctors deaths were directly related to their professional work.

Based on available information excessive doctors deaths during COVID-19 pandemics were strongly related to not adequate personal security in the first phase of pandemics, overwork, very stressful work conditions and also due to ill health status of many middle-aged and older physicians [34,35].

The more extended discussion about the reasons of doctors death during COVID-19, relationship between vaccination rate and deaths, adequate protection of medical personnel in the worldwide scale and in particular countries deserve separate analysis and is out of scope of this publication.

### Violence against healthcare workers

Although violent behaviour toward health professionals has been reported earlier in various parts of the world, the phenomenon seems to be increasing in frequency in the current century [36–38].

Scientific publications indicate that medical staff around the world are suffering non-physical (insults, threats, bullying, stalking) and physical aggression including sexual assaults or even murder by patients, their family members or visitors. Regardless of the region of the world, tragic information about physicians' death resulting from physical assaults appear in both scientific and mass-media reports [39–43].

World literature indicates that the targets of acts of violence are not only physicians but also nurses, midwives, paramedics and even security officers [44–50]. It seems that healthcare workers in the area of surgery, psychiatry/mental health, intensive care and emergency medicine are at the highest risk of experience of violence [41,44,46,47,51–55]. The most alarming reports concerning aggression directed towards medical staff come from China, where the incidence of violence has increased by about 11% each year since 2000 [56–59].

Chen et al. found that almost 90% of physicians experienced verbal violence and >10% experienced physical one [59]. The situation in other regions of the world is also concerning. The research conducted in Palestinian hospitals showed that the majority of emergency departments personnel were exposed to workplace violence: 69.8% to verbal abuses, 48.4% to threats, 8.6% to sexual harassments and 35.6% to physical assaults [46]. These findings are in line with studies from other countries. For example, in an Iraqi study, 85% physicians experienced nonphysical violence and 14–31% experienced physical one [60,61]. Similarly, Hasan et al. reported that >80% of participants experienced verbal assault, 13.5% of physical assault and 2% of sexual assault [55]. A Jordanian study revealed violence exposure in case of 63% doctors (verbal abuse – 56%, nearly 6% – physical abuse and another 6% for emotional abuse) [62]. In Türkiye 39–78% physicians, depending on the department, admitted to experience violence in the year preceding the survey [47,54]. Similarly, in a 2018 Pakistani study, 38.7% of physicians experienced verbal violence whereas 15.3% physical violence in the pursuit of their occupational duties [63].

The issue of violence against medical staff is not limited to Asian region with important reports concerning this issue also coming from Mexico and the USA [50,64,65].

Finally, there are some studies conducted in European countries. According to the Italian studies over a half of healthcare workers experienced non-physical and 4–28% physical assault over the past year [66,67]. British reports suggest that 28–35% healthcare workers experienced non-physical violence and 14–19% experienced physical assault during the previous year [49,68]. Publication by Johansen et al. [69] shows that in 2014 almost 24% of Norwegian doctors were exposed to real act of violence at least once. In turn, the Spanish study showed that 53% physicians are at high risk of violence and ≤5% experienced violence with physical assault [70].

Of note, some studies emphasize the fact that the frequency of violence against medical professionals has been observed to be steadily increasing in recent years [49,54,60,69].

### Sudden unexpected physicians' deaths during professional duties in Poland

In Polish medical scientific journals, as well in other reliable sources, the authors were unable to find any papers or report concerning the sudden unexpected death of medical doctors or other medical staff. Moreover, no official data on sudden deaths of physicians at work were found. So far, the only source of information about these cases in Poland are personal communication and media reports.

In 2025 Anna Z., a 39-year-old surgeon, who worked at the Specialist Hospital in Radom, had fallen asleep after her duty and did not wake up [71]. A similar case involved a 40-year-old urologist at Pabianickie Medical Center, who fell asleep while on duty and died [72].

Another 39-year-old anaesthesiologist died in hospital, he used to work around 100 h/week [73]. Similarly, another 39-year-old cardiologist died while on weekend duty, whose body was found on Monday around 9 a.m. in the doctors' room [74]. In another case, a 59-year-old surgeon from Włoszczowa died after a 24-hour duty [75]. The hospital doctors tried to save him, but he died on the operating table.

It later came to light that he was supposed to restart work at 3 p.m. the same day in the Emergency Department.

Moreover, a young medical doctor in Niepołomice fainted while on night duty from 6 p.m. to 8 a.m. Resuscitation was performed, but she could not be saved and died [76]. Another example of work overload came to light when a 44-year-old anaesthesiologist in Białogard died after 4 days and nights of duty [77].

More examples of physicians' deaths can be cited after long duties, sometimes lasting >72 h without a break. Accordi ng to the European Union directive, a doctor should not work >48 h/week. However, it is unrealistic in Poland, as some parts of the hospital would need to be closed to fulfil this requirement because there are not enough doctors. Staff shortage in Polish hospitals made the situation worse, with some doctors and nurses working in multiple healthcare institutions without informing their primary employers. A new phenomenon in Poland is the brutal aggression from patients towards physicians, nurses and paramedics, sometimes resulted in the tragic death of medical professionals. A 47-year-old orthopaedist from Kraków was murdered by a patient who was dissatisfied with his treatment [78].

### DISCUSSION

This review shows that physicians in many countries are at risk of sudden death while performing their occupational duties. The alarming situation for doctors includes incidents of sudden deaths resulting from extreme workload, burnout, suicidal ideations, and violence. The most frequently indicated reasons of physicians' sudden unexpected death during professional duties are prolonged working hours, excessive supervisor expectations and insufficient or lack of awareness and support systems. Several specialties, including anaesthesiology, surgery, psychiatry, intensive care and emergency medicine, carry higher exposure due to workload and pressure.

Behind the phenomenon of unexpected physicians' deaths, there is not only individual tragedy but also a dangerous medical and social issue influencing the safety of patients. Staff shortages, multiple employment contracts and limited rest time add further risks for both physicians and patients. An exhausted doctor not only poses danger to himself, but also to the patients. There are some strategies which could help like: education and prevention, psychological support, reduction in duty hours and better remuneration and work conditions.

The problem is visible all over the world, however, the scale of the problem is different and evidence suggests that regulated working hours and better staffing can reduce risk. A number of publications have emphasized the need to implement international working standards including following standard working hours, self-awareness, screening tests, psychological support, security and fair compensation [15,21,63,79].

The global literature on this topic remains limited and a major gap is the lack of systematic reporting at institutional and country levels. This also represents a constraint for this paper. In many settings, sudden physician deaths are known only through media or informal accounts, which limits understanding and delays action.

Addressing these needs requires coordinated action from governments, medical societies and healthcare organisations to build stronger data systems, implement targeted prevention strategies, improve working conditions, protect physicians and strengthen patient safety. Achieving this requires further research and the development of both theoretical and practical applications of new findings.

The main strength of this paper is comprehensive description of sudden unexpected doctors deaths during performing professional duties as neglected but very important public health problem. Because of the scarcity of scientific publications and reliable official reports that the authors included also some media reports from reputable sources. The authors intend not only to present examples from many countries with different health systems, work culture and economic conditioning but also to describe the main reasons of unexpected doctors deaths as well as to describe some protective measurements.

There are certainly some limitations of this paper. The authors included only papers and reports published in English and in Polish language, thus publications and reports in other languages or published in scientific journals not indexed in international data bases could be overriden. Due to limited volume of the review it was not possible to present comprehensive discussion about existing policies and measurements existing in several countries in order to reduce physicians burnout, overwork or mobbing. This important issues will be described in the next publications.

## CONCLUSIONS

Sudden unexpected doctors death during performing professional activities is frequently neglected in medical literature but not rare global phenomenon. Sudden doctors deaths due to suicides or violence are reported in young, middle-aged and older doctors. Among most important reasons of sudden doctors' death are very stressful work, overwork, burnout, excessive supervisor expectations, ill health and violence.

Based on comprehensive literature overview it seems that these tragic events are more frequent in countries with not adequate legal regulations related to doctors work, as well in countries with shortage of medical personnel and not adequate renumeration of medical staff. Some medical specialties, like anaesthesiology, surgery, intensive care, emergency medicine, psychiatry carry greater risk due to workload and excessive pressure.

Addressing the needs to reduce significantly the numbers of doctors death on duty requires coordinated action from governments, medical societies and healthcare organisations to build stronger data systems, implement targeted prevention strategies, improve working conditions, protect physicians and strengthen patient safety. Achieving this requires further research and the development of both theoretical and practical applications of new findings.
